# ﻿The intraspecific variation of morphology and coloration of field crickets: a taxonomic revision of Chinese *Gymnogryllus* Saussure, 1877 and *Phonarellus* Gorochov, 1983 (Orthoptera, Gryllidae, Gryllini)

**DOI:** 10.3897/zookeys.1129.87706

**Published:** 2022-11-15

**Authors:** Ning Wang, Huateng Huang, Li-Bin Ma

**Affiliations:** 1 College of Life Sciences, Shaanxi Normal University, Xi’an, China, 710119 Shaanxi Normal University Xi’an China

**Keywords:** Cricket, genitalia, Grylloidea, morphological diversity, new synonym, taxonomy

## Abstract

After extensive sampling of specimens from species found in China, we examined the intraspecific morphological variation of several characters used for species delimitation in two closely related cricket genera, *Gymnogryllus* Saussure, 1877 and *Phonarellus* Gorochov, 1983. We found that the characters (male genitalia in *Gymnogryllusodonopetalus* Xie & Zheng, 2003 and *Phonarellusritsemae* (Saussure, 1877), and coloration of the hind leg in *Phonarellusminor* (Chopard, 1959)) exhibit considerable amounts of variation within species, and are thus not reliable characters for species differentiation. Therefore, we revised the taxonomy of these two genera. Five synonyms are proposed: *G.yunnanensis* (= *G.odonopetalus*) **syn. nov.**, *G.striatus* (= *G.odonopetalus*) **syn. nov.**, *G.longus* (= *G.odonopetalus*) **syn. nov.**, *G.tumidulus* (= *G.odonopetalus*) **syn. nov.**, and *P.flavipes* (= *P.minor*) **syn. nov.** All species mentioned above are described and illustrated. Keys and a distribution map are provided.

## ﻿Introduction

*Gymnogryllus* Saussure, 1877 and *Phonarellus* Gorochov, 1983 have species in China that are difficult to distinguish based on morphology, and we found that some of the “different” species co-occur at the same collection site and at the same time. Many of these species were proposed based on a limited number of specimens (e.g., Xia et al. 1991; Ma and Zhang 2011). That meant some of the characteristics for species delimitation in the previous taxonomic literature might reflect diversity within species rather than differences between species. The validity of these species needed re-examination.

*Gymnogryllus* was established by Saussure in 1877 with *Grylluselegans* Guérin-Méneville, 1834 as the type species. It is distinguished from other genera of the tribe Gryllini by the face (distinctly longer than wide), oblique veins (slightly curved), and the ovipositor (short and armed with a small hook in the anterior of the lower valvae). Species of this genus have a similar appearance, and the male genitalia features are the primary characteristics for species identification. Currently, 45 species are reported worldwide, from India to Australia, and most of them are found in tropical Southeast Asia (Indian subcontinent, western Himalayas, Burma, Vietnam, and Malaysia) (Cigliano et al. 2022). In China, there are eight *Gymnogryllus* species. *Gymnogrylluscontractus* Liu et al., 1995 and *Gymnogryllusodonopetalus* Xie & Zheng, 2003 were described in the 20^th^ century. In 2011, an additional six Chinese species were reported (*Gymnogryllusdolichodens* Ma & Zhang, 2011, *Gymnogrylluslongus* Ma & Zhang, 2011, *Gymnogryllustumidulus* Ma & Zhang, 2011, *Gymnogryllusextrarius* Ma & Zhang, 2011, *Gymnogryllusyunnanensis* Ma & Zhang, 2011, and *Gymnogryllusstriatus* Ma & Zhang, 2011). These six Chinese species are distributed in the same province and are similar in body size and forewing morphology, for example, inclined rectangular mirror and internal dividing vein with three branches. The only characteristic for identifying these species is the posterior angle of the epiphallus. Here, we examined whether this diagnostic feature is stable by using multiple specimens per species to assess the amount of intraspecific variation.

**Figure 1.**
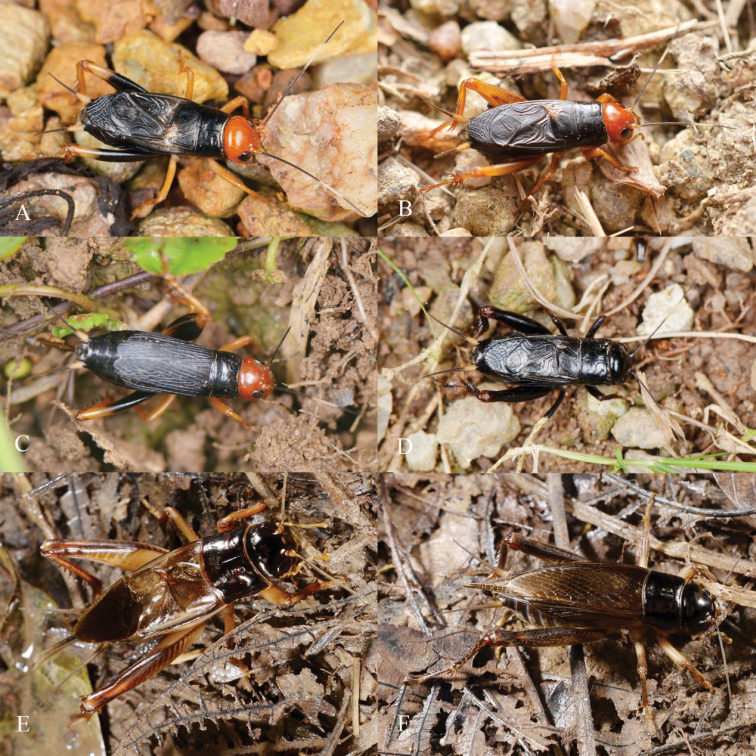
Living photos of some *Phonarellus* and *Gymnogryllus* species **A, B** male of *P.minor* (with varing proportion of black areas on hind legs) **C** female of *P.minor***D** male of *P.ritsemae***E** male of *G.odonopetalus***F** female of *G.odonopetalus* (Photos **A–D** were provided by Zhang, Tao, and **E, F** were photographed by He, Zhixin).

Gorochov established the genus *Phonarellus* for species originally belonging to *Gymnogryllus* and designated *Gymnogryllusminor* Chopard, 1959 as the type species (Gorochov, 1983). Compared to *Gymnogryllus*, species of *Phonarellus* are smaller, the cercus base is of light color, and the apical area of the genitalia is obviously different between both genera. We recognize the genus by its contrasting coloration of antennae (proximal segments colored light and most of the hind portion colored dark, sometimes completely white), ocelli almost arranged in a line, shiny and smooth pronotum, and somewhat leathery elytra (Fig. 1). The genus contains 16 species and they are distributed from Africa to the Indo-Malayan region (Afghanistan, Bangladesh, Burkina Faso, China, Gabon, India, Japan, Kenya, Mali, Sierre Leone, and Vietnam) (Cigliano et al. 2022). Four *Phonarellus* species are recorded from China (*Phonarellusritsemae* (Saussure, 1877), *Phonarellusminor* (Chopard, 1959), *Phonarellusflavipes* Xia, Liu & Yin, 1991, and *Phonarelluszebripe* He, 2022). Two species, *P.minor* and *P.flavipes*, occur in Yunnan and Guangdong and are of very similar appearance. The only difference between *P.minor* and *P.flavipes* is that the posterior femora of *P.flavipes* have no black area and the markedly separated first and second oblique veins at the base (Xia et al. 1991). However, the proportion of the black area varies even within *P.minor*. Thus, classification based only on this feature is questionable.

**Figure 2.**
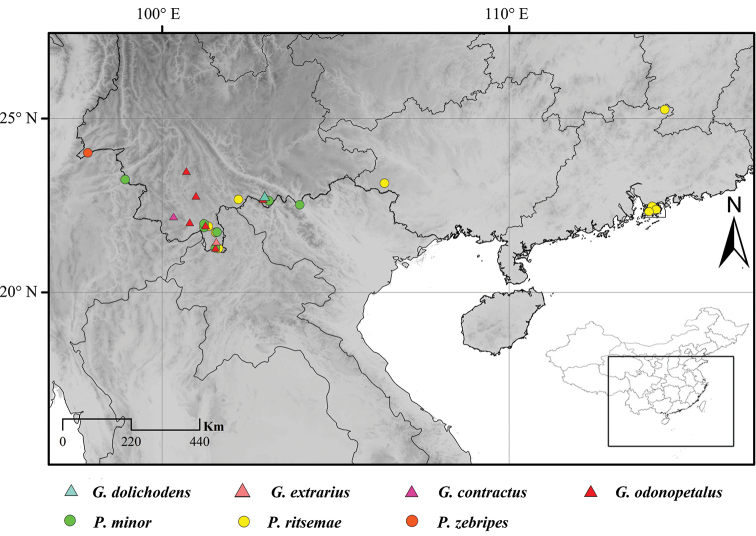
Distributions of *Phonarellus* and *Gymnogryllus* in China (Note: the locality of *P.zebripes* cited from Liu and He (2022), and the remaining location information is based on the data of examined materials).

To address the problems with species of Chinese *Gymnogryllus* and *Phonarellus*, we collected more than 100 specimens (35 specimens of *Gymnogryllus* and 130 specimens of *Phonarellus*) and examined their morphological characteristics. Based on our results, we consider that four species in *Gymnogryllus* are junior synonyms of *G.odonopetalus*, and *P.flavipes* is a junior synonym of *P.minor*. The diagnostic characteristics previously proposed for species identification are unreliable because of extensive variation. New checklists of Chinese *Gymnogryllus* and *Phonarellus* species, with keys to species and distribution maps (Fig. 2), are provided.

## ﻿Materials and methods

### ﻿Specimens and photographs

Most specimens were attracted to a high-pressure mercury lamp (500W) in the field. The specimens were preserved in analytical-grade ethanol during fieldwork and then pinned and dry-preserved in laboratory. After softening, dissecting needles were used to pull out the male genitalia from the gonopore. The dissected genitalia complexes were prepared by placing them into a concentrated solution of alkaline protease (0.2 g/ml, AOBOX, China) with a water bath temperature of 40–50 °C for 48 hours. Identification of involved species is mainly based on male morphology. Whole bodies were photographed with a VHX-6000 digital microscope (Keyence, Osaka, Japan). Figures of genitalia and body details were produced using a ToupCam Digital camera and bundled software (ToupTek, Hangzhou, China).

### ﻿Measurements and abbreviations

All specimens were measured using ToupCam Digital camera (E3ISPM05000KPA) and bundled software (ToupTek, Hangzhou, China). All the measurements are in millimeters (mm). Nomenclature of male genitalia follows Gorochov (1996) and Ma and Zhang (2011) and measurement abbreviations are as below: **BL** body length (from head to tip of abdomen); **HW** head width; **EW** eye width; **PL** pronotum length; **PW** pronotum width (max. width of pronotum); **FWL** forewing length; **HWL** hind wing length (length of uncovered part); **DVL** length of dialogue vein; **ML** mirror length (from fore to hind margin); **CL** cercus length; **FTL** fore tibiae length; **TTL** length of tibial tympanum; **MTL** middle tibiae length; **HLL** hind femur length; **HTL** hind tibiae length.

### ﻿Proportion of colored area on posterior femora of *Phonarellusminor*

We used 42 *P.minor* specimens with at least one intact hind leg. Photos of these posterior femora were taken with a VHX-6000 digital microscope (Keyence, Osaka, Japan) and processed in ImageJ. We used the Threshold function in ImageJ ver.1.53k (Schneider et al. 2012) software to obtain the size of the posterior femora and the size of the black area on each photographed hind leg. The proportion of black area (the ratio between the size of the black area and the total area) is calculated, and the distribution is drawn in Microsoft Excel (Microsoft Office 2016).

### ﻿Distance between the base of the first and second oblique veins of *P.minor*

We used a ToupCam Digital camera (E3ISPM05000KPA) and bundled software (ToupTek, Hangzhou, China) to measure the distances between the first and second oblique veins at the base of 42 specimens. The distributions were graphed in Microsoft Excel (Microsoft Office 2016).

Acronyms used for the institutions where those examined materials are deposited:

**SNNU**Museum of Flora and Fauna of Shaanxi Normal University, Xi’an, China;

**NWAFU** Entomological Museum of Northwest A&F University, Yangling, China;

**SEM** (IEAS) Shanghai Entomological Museum, CAS, Shanghai, China.

## ﻿Taxonomy

### ﻿Checklist of Chinese *Gymnogryllus* and *Phonarellus* species

**Genus *Gymnogryllus* Saussure, 1877**

***Gymnogrylluscontractus* Liu, Yin & Liu, 1995**

**Chinese name.** 狭膜裸蟋

**Distribution.** Yunnan.

***Gymnogryllusodonopetalus* Xie & Zheng, 2003**

***Gymnogryllusyunnanensis* Ma & Zhang, 2011, syn. nov.**

***Gymnogryllusstriatus* Ma & Zhang, 2011, syn. nov.**

***Gymnogrylluslongus* Ma & Zhang, 2011, syn. nov.**

***Gymnogryllustumidulus* Ma & Zhang, 2011, syn. nov.**

**Chinese name.** 齿瓣裸蟋

**Distribution.** Yunnan, Guangxi, Guangdong.

***Gymnogryllusdolichodens* Ma & Zhang, 2011**

**Chinese name.** 长突裸蟋

**Distribution.** Yunnan.

***Gymnogryllusextrarius* Ma & Zhang, 2011**

**Chinese name.** 外突裸蟋

**Distribution.** Yunnan.

**Genus *Phonarellus* Gorochov, 1983**

***Phonarellusminor* (Chopard, 1959)**

***Phonarellusflavipes* Xia, Liu & Yin, 1991, syn. nov.**

**Chinese name.** 小音蟋

**Distribution.** Guangxi, Hainan, Guangdong, Yunnan.

***Phonarellusritsemae* (Saussure, 1877)**

**Chinese name.** 利特音蟋

**Distribution.** Guangxi, Zhejiang, Yunnan, Guangdong, Hong Kong.

***Phonarelluszebripes* He, 2022**

**Chinese name.** 斑腿音蟋

**Distribution.** Yunnan.

### ﻿Species accounts

**Orthoptera: Grylloidea; Gryllidae; Gryllinae**

#### ﻿Genus Gymnogryllus

Taxon classificationAnimaliaOrthopteraGryllidae

Saussure, 1877

1300E0C5-9285-53CC-8D7D-A8DB4711C8BE

Gymnogryllus Brunner von Wattenwyl 1893: 197; Gorochov 1983: 321; Yin and Liu 1995: 194; Ma and Zhang 2011: 31; Gorochov 2022: 4.
Brachytrypus (Gymnogryllus) Saussure, 1877: 291.

##### Type species.

*Grylluselegans* (= *Gymnogryllusleucostictus*). Brachytrypus (Gymnogryllus) Saussure, 1877: 291.

##### Distribution.

India, Australia, western Himalayas, Burma, Vietnam, Malaysia, China.

##### Diagnosis.

Body large. Head, pronotum and much of hind femur blackish brown; rest of body of light color. Light brown bands uniformly distributed over posterior peduncle. Forewings not reaching tip of abdomen; hind wings largely surpassing abdomen. Mirror inclined rectangular. The length of the apical field of forewings varies among individuals. Subgenital plate shaped as hook. Genitalia large, in caudal view, epiphallus arch-shaped and the apically armed with a pair of long teeth. The space between the teeth and the shape of them varied among individuals (Fig. 5). Ovipositor very short, almost straight.

##### Remarks.

Eight species of *Gymnogryllus* are reported from China, and six of them have been found in Yunnan. Among them, *G.longus*, *G.tumidulus*, *G.yunnanensis*, and *G.striatus* have been described for differences in the angle of the epiphallic apex and the length of the apical field of tegmen. However, they are similar to *G.odonopetalus* in appearance and can be collected from the same location at the same time. We compared specimens collected from the same site and concluded that these two features present intraspecific variation and are unreliable for species delimitation. We consider that all four taxa are synonyms of *G.odonopetalus*.

### ﻿Key to *Gymnogryllus* species in China

**Table:** 

1	Mirror narrow	***G* . *contractus***
–	Mirror much broad	**2**
2	Forewings extended to tip of abdomen	***G* . *dolichodens***
–	Forewings not extended to tip of abdomen	**3**
3	Epistomal suture curved upward medially, frons with light angular patch	***G* . *odonopetalus***
–	Epistomal suture straight, frons uniform colored	***G* . *extrarius***

#### ﻿ Gymnogryllus odonopetalus

Taxon classificationAnimaliaOrthopteraGryllidae

Xie & Zheng, 2003

B188F7B0-6336-55AD-91F9-1F3BA1430361

**Figure 3.**
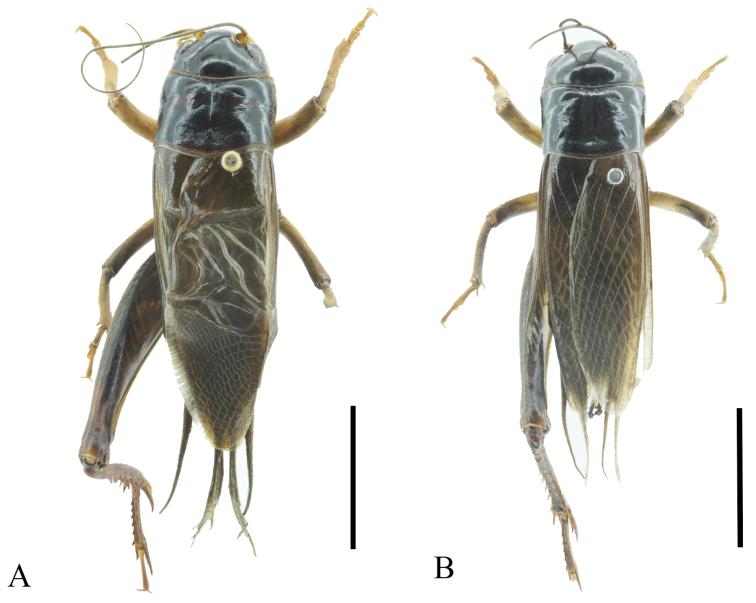
Bodies of *G.odonopetalus***A** male **B** female. Scale bar: 10 mm.

**Figure 4.**
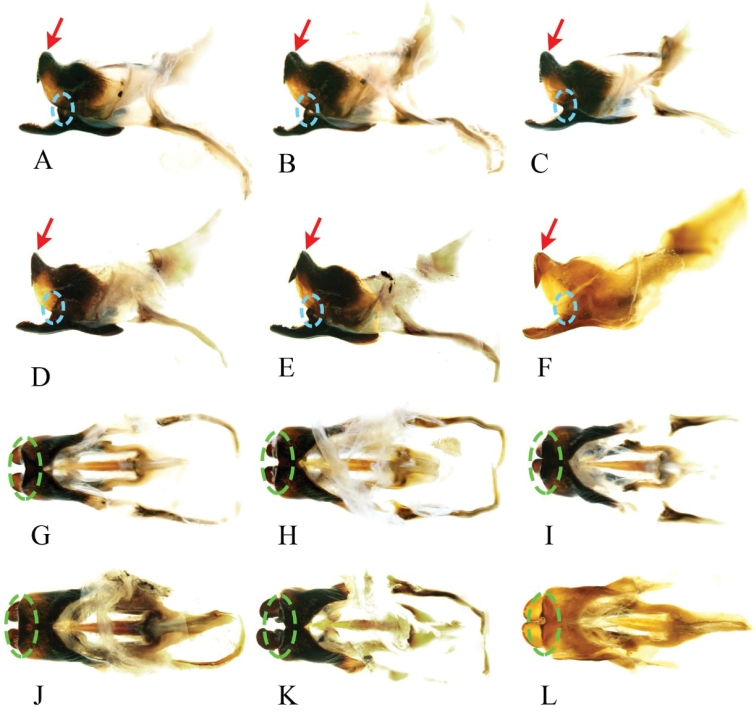
Intraspecific variation of genitalia in *G.odonopetalus***A–F** genitalia from six individuals in lateral view (Note: the red arrows point to the posterior of epiphallus, highlighting variations among individuals; the blue circles indicate the protuberance of mid-ectoparamere) **G–L** genitalia of the same six individuals in dorsal view (Note: the green circles indicate the median lobe of the epiphallus posterior).

**Figure 5.**
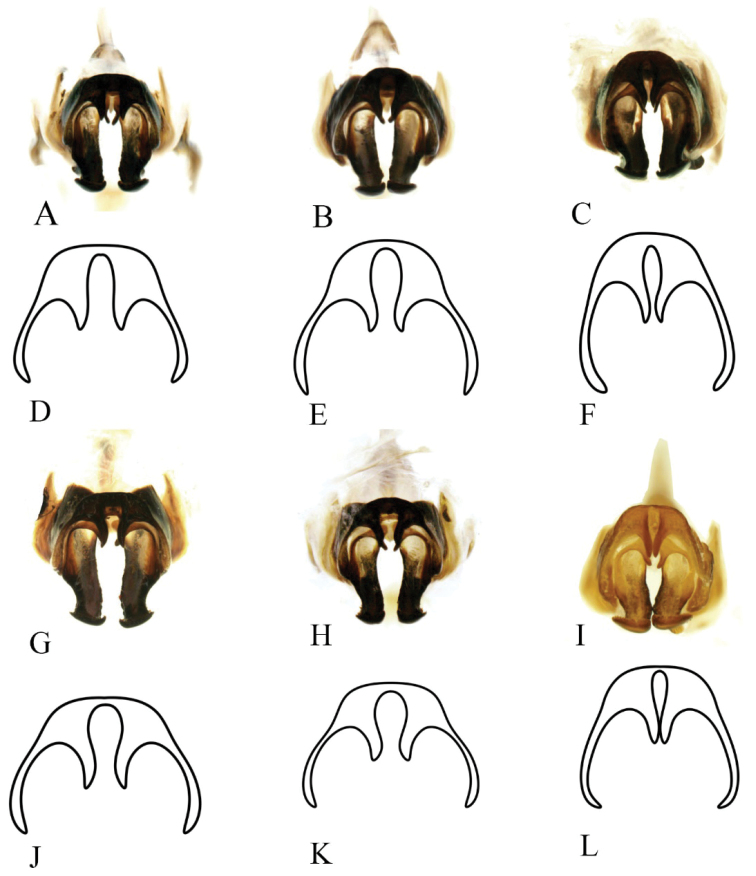
Intraspecific variation of genitalia in *G.odonopetalus***A–C** and **G–I** photos of genitalia from six individuals in caudal view **D–F** and **J–L** drawings of genitalia from the same six individuals in caudal view.

Figs 1E–F , 3 , 4 , 5

Gymnogryllus odonopetalus Xie & Zheng, 2003: 496, 498.
Gymnogryllus yunnanensis Ma & Zhang, 2011: 31–40, syn. nov.
Gymnogryllus longus Ma & Zhang, 2011: 31–40, syn. nov.
Gymnogryllus tumidulus Ma & Zhang, 2011: 31–40, syn. nov.
Gymnogryllus striatus Ma & Zhang, 2011: 31–40, syn. nov.

##### Holotype.

Type locality: Menglun, Xishuangbanna, Yunnan, China. Deposited at Museum of Flora and Fauna of Shaanxi Normal University, Xi^’^an, China (SNNU).

##### Specimens examined.

**China**: 1 male (holotype), Yunnan, Xishuangbanna, Menglun, Sept. 8, 1999, Xie, Lingde coll. (SNNU); 2 males and 1 female, Yunnan, Honghe, Wengdang, Jun. 11, 2009, Ma, Libin coll. (SNNU); 1 female, Yunnan, Mengla, Shangyong, Longmen, May 13, 2013, Ma, Libin coll. (SNNU); 1 male, Yunnan, Mengla (or Wangtianshu), Oct. 2, 2014, Zhang, Tao coll. (SNNU); 3 males, Yunnan, Jinghong, Jul. 11, 2018, Peng, Zhong coll. (SNNU); 2 males, Yunnan, Pu’er, Jinggu, Aug. 17, 2021, He, Zhixin coll. (SNNU); 7 males, Yunnan, Pu’er, Simaoqu, Aug. 18, 2021, He, Zhixin coll. (SNNU); 15 males, Yunnan, Mengla, Menglun, Aug. 25, 2021, He, Zhixin coll. (SNNU); 3 females, Yunnan, Mengla, Menglun, Aug. 25, 2021, He, Zhixin coll. (SNNU).

##### Distribution.

(Fig. 2). China (Yunnan, Guangxi, Guangdong).

##### Measurements (mm).

**Male (*N* = 30)**: BL 27.73–29.42; HW 6.42–6.84; PL 4.86–5.32; FWL 18.62–21.32; HLL 16.27–17.12; HTL 9.43–10.14; EW 1.56–1.79; PW 7.62–7.98; HWL 6.04–6.84; DVL 4.62–4.96; ML 3.72–3.98; CL 8.15–8.54; FTL 5.42–5.76; MTL 6.29–6.45.

##### Description.

***Male*** (Figs 1E, 3A). Head longer than wide, as wide as fore margin of pronotum. Vertex smooth and broad. Occiput slightly inclined. Frontal rostrum as wide as one eye and flattened. Scape of antennae flat, shield-like, and as wide as half of frontal rostrum. With three small ocelli, arranged in a straight line, median ocellus semilunar. Eyes about 1/4 length of head. Epistomal suture straight and close to eyes. Labrum slightly convex and rhombus shaped. Last segment of maxillary palpi slightly narrower than the third. Labial palpi the middle segment longer than the others.

Pronotum disc rectanglur, anterior margin concave, posterior margin sinuated; a longitudinal groove in middle of pronotum, about 2/3 the length of the pronotum. Fore margin of pronotum rough and densely pubescent. Tegmen reaching tip of abdomen; with three oblique veins, outmost nearly vertical and straight, two internal inclined and longer than outmost; and them converging diagonal vein. Diagonal vein curved and anteriorly forked. Chord veins three, the internal two extremely curved, connected at bottom. Between diagonal vein and the most internal chord vein armed with two transverse veins. Mirror large, inclined rectangular. Apical field triangular, about 2/5 the length of tegmina, variable among individuals, armed with rectangular cells.

Fore tibiae with inner and outer tympanum, inner tympanum small and ovoid, outer longer-oval. Hind femora brown with light stripes. Distal of hind tibiae with five dorsal spurs on both sides; apical spurs six, the inner apical spurs three (the dorsal one longest, the ventral one shortest and 1/4 length of the longest one, the middle one about 2/3 length of the longest), and the outer apical spurs three (equal length of the dorsal one and the ventral one, about 2/3 length of the middle one). Subgenital plate hook-like. Cercus straight and short; with long hair sparse and short hair dense.

***Genitalia*** (Figs 4, 5). Male genitalia robust, epiphallus arch-shaped in front view; inner side of medial lobe armed with a pair of teeth. Median notch between paired apical teeth and length of teeth varyable between individuals. End of middle lobes of epiphallus bent upward, for less than 90°, varyable between individuals. Notch of epiphallus anterior variable, with bottom angular or broad and arc-shaped. Outer edge of end of ectoparamere armed with irregular numbers of teeth.

***Coloration*** (Fig. 3). Head, pronotum and much of the hind femur blackish brown. Occiput uniformly blackish brown. 1/3 part of cheek bottom brown. Frons black, ventral quarter of lateral lobe with a light stripe. Hind femur black.

**Female** (Figs 1F, 3B). Slightly smaller than male, with tegmina dark brown. Ovipositor as long as pronotum.

##### Remarks.

Ma and Zhang (2011) proposed four species, *G.yunnanensis*, *G.striatus*, *G.longus*, and *G.tumidulus*, based on the angle between the apical epiphallic teeth, the posterior edge of the medial lobe of epiphallus, and the apical field of the forewing. We examined our specimens and determined that these characters present intraspecific variation. In particular, the angle between the apical teeth of epiphallus shows tremendous variation between specimens (Fig. 4A–F). Besides, the epiphalli of these four species are armed with two pairs of teeth inside, which is consistent with the holotype of *G.odonopetalus* deposited in SNNU (in fact, we found that one pair of teeth is formed by the ectoparamere curved at the tip on the type specimen of *G.odonopetalus*). Therefore, we regard *G.yunnanensis*, *G.striatus*, *G.longus*, and *G.tumidulus* as junior synonyms of *G.odonopetalus*.

Hence, the species *G.odonopetalus* has some charecters showing intraspecific variation even within specimens collected from the same place and time. In lateral view, the angle between the apical teeth and the posterior edge of the medial lobe of epiphallus is variable among individuals (red arrows in Fig. 4); the protuberances in the middle of the ectoparamere vary in size (blue circles indicate the protuberance of mid-ectoparamere in Fig. 4). In dorsal view, the end of the epiphallus is variable (shown by green circles in Fig. 4G–L); the anterior notch of epiphallus is morphologically also diverse. In caudal view, shape and space between apical teeth of epiphallus are variable (drawing of epiphallus in Fig. 5D–F, J–L); the length of the apical area of the ectoparamere varies among individuals.

#### ﻿Genus Phonarellus

Taxon classificationAnimaliaOrthopteraGryllidae

Gorochov, 1983

11AD3131-B55B-515E-8C0F-25DDBCC7F17D

Phonarellus Gorochov, 1983: 323; Yin and Liu 1995: 194; Ma and Zhang 2011: 31.

##### Type species.

*Gymnogryllusminor*.

##### Distribution.

Afghanistan, Bangladesh, Burkina Faso, China, Gabon, India, Japan, Kenya, Mali, Sierre Leone, Vietnam.

##### Diagnosis.

Ocelli positioned in an almost straight line. Antenna ornamented with white ring-like pattern. Scapus conspicuously narrower than the half-width of the rostrum. Apical field shorter than mirror or slightly longer. Both tympana present. Hind tibiae shorter than half the length of hind femur. Epiphallus with large lateral lobes but without median lobe. Cerci usually dark with light proximally. We regard all species as belonging to *Phonarellus*, whose characters are as follows: ovipositor well developed and rather long; ectoparamere short.

##### Remarks.

Four species of this genus have been reported from China (*P.ritsemae*, *P.minor*, *P.flavipes*, and *P.zebripes*). *Phonarellusflavipes* has been described for its yellow hind legs and the interval between the anterior of the first and second oblique veins. But these characters can also be found in *P.minor* living side by side with *P.flavipes*. Studying a large number of specimens of these three taxa from Yunnan and Guangdong, we tested whether the color of hind legs is a valid trait for species delimitation, and provided a description of *P.ritsemae*.

### ﻿Key to *Phonarellus* species in China

**Table:** 

1	Body bicolored, head reddish with most of the remainder dark	***P.minor***
–	Body almost uniformly dark	**2**
2	Hind femora uniformly dark	***P.ritsemae***
–	Hind femora black and white	***P* . *zebripes***

#### ﻿ Phonarellus minor

Taxon classificationAnimaliaOrthopteraGryllidae

(Chopard, 1959)

DDCBC5BC-25BA-5527-81C5-DC60A4AB005B

**Figure 6.**
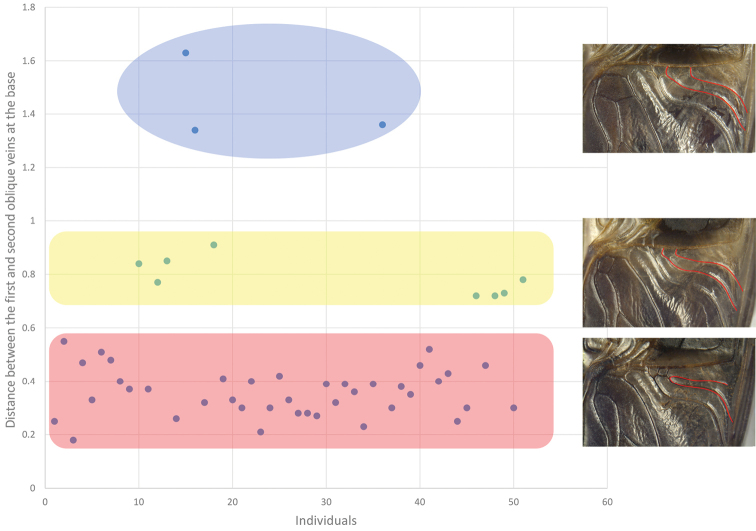
Distance between the first and second oblique vein of *P.minor* (Note: The data in the graph were measured from 42 samples. All the data can be roughly divided into three groups: distance less than 0.6 mm, distance greater than 1.2 mm, and distance between 0.7 mm and 1 mm, among which, individuals with distance less than 0.6 mm are the majority. The figure of the veins on the right, from top to bottom, represents the maximum, median and minimum distances, respectively. Units: mm).

**Figure 7.**
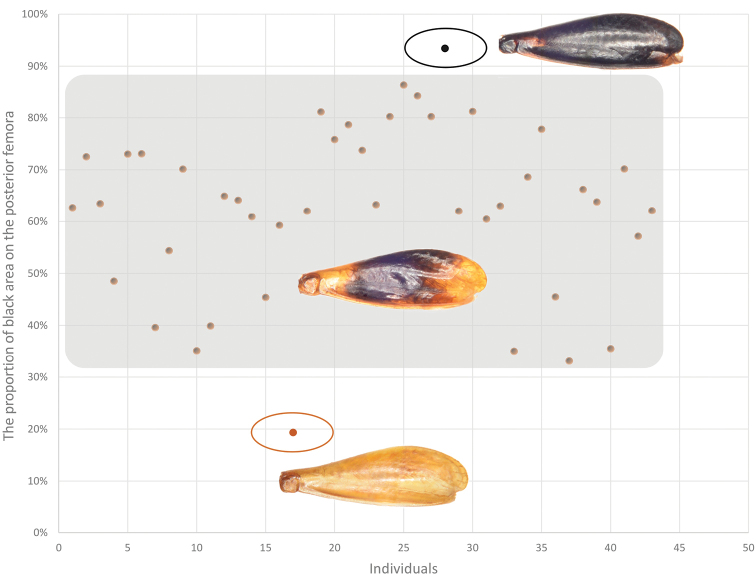
This figure shows that the distribution of the area-to-total area ratio of the black area of *P.minor* (Note: The data in the graph were measured from 42 samples. In the figure, miscellaneous colors, i.e., individuals with black proportions between 30% and 90%, accounted for the majority, while light-colored individuals, i.e., individuals with black proportions no higher than 20%, and black individuals, i.e., individuals with black proportions higher than 90%, were very few and only one in our sampling respectively.).

**Figure 8.**
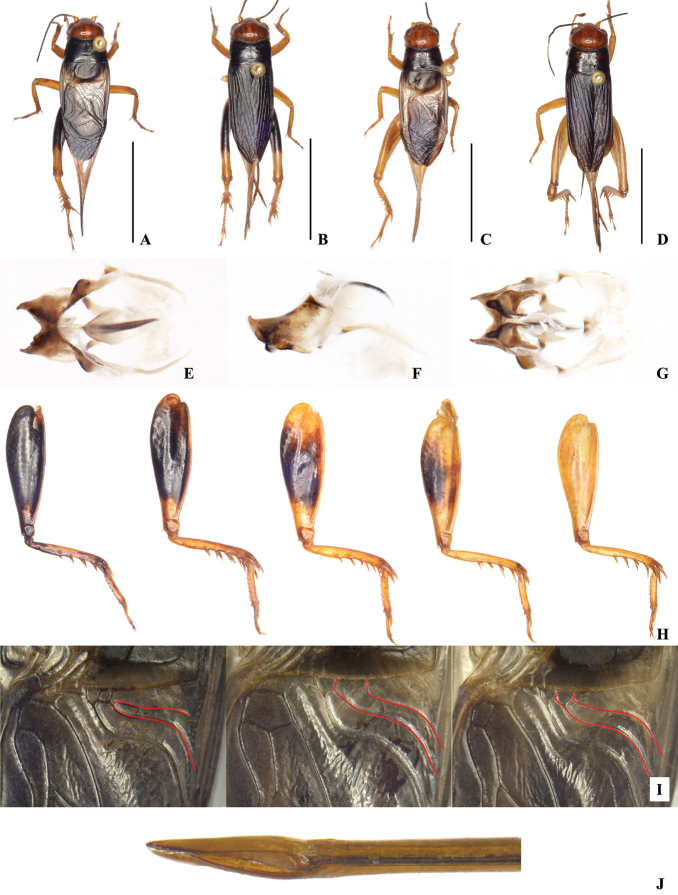
*Phonarellusminor* (Chopard, 1959) (**A–J**) **A–D** bodies of *P.minor* (**A, C** males **B, D** females; scale bar: 10mm **A, B** specimens with dark colored hind legs **C, D** specimens with light-colored hind legs) **E–G** genitalia (**E** dorsally viewed **F** laterally viewed **G** ventrally viewed) **H** color variation of hind legs from dark to light **I** variable intervals at the base of oblique veins (Note: The red line point to the first and second oblique veins. The distance between the two veins in the rightmost figure is the smallest (see Fig. 6, i.e., less than 0.6 mm), and the sampled individual has black hind legs (see Fig. 7, i.e., the percentage of black is higher than 90%); the distance between the two veins in the middle figure is the largest (see Fig. 6, i.e., greater than 1.2 mm), and the sampled individual has miscellaneous colored hind legs (see Fig. 7, i.e., 30%–90% black); the distance between the two veins in the rightmost figure is medium (Fig. 6, i.e. 0.7mm-1mm), and the hind legs of the sampled individuals are light colored (Fig. 7, i.e. the percentage of black is less than 20%). Combining the information in Figs 6, 7, it can be inferred that the change in veins distance does not correlate entirely with the variation in the color of the hind legs) **J** ovipositor.

Figs 1A–C , 6 , 7 , 8

Gymnogryllus minor Chopard, 1959: 1; Bhowmik 1985: 14.
Gymnogryllus kashmirensis Bhowmik, 1977: 24, misidentification.
Phonarellus (Phonarellus) minor : Gorochov 1983: 91, 323–328; Kim and Pham 2014: 61; Gu et al. 2018: 11.
Phonarellus minor : Yin and Liu 1995: 138–139; Saeed et al. 2000: 176; Xie 2004: 116
Gymnogryllus (Phonarellus) minor : Ingrisch and Garai 2001: 759.
Phonarellus flavipes Xia et al., 1991: 123; Yin and Liu 1995: 49, syn. nov.

##### Holotype information.

Type locality: Asia-Tropical, Indian Subcontinent, India, Kerala, Malabar Coast, Mahé. Deposited at Muséum National d’Histoire Naturelle, Paris, France (not examined).

##### Specimens examined.

**China**: 36 males and 28 females, Yunnan, Mengla, Shangyong, Longmen, 1030 m, May 13, 2013, Ma, Libin coll. (SNNU); 5 females, same location as before, 1030 m, May 18, 2013, Ma, Libin coll. (SNNU); 4 males and 4 females, same location as before, 943 m, May 13, 2013, Ma, Libin coll. (SNNU); 3 males, same location as before, 996 m, May 13, 2013, Ma, Libin coll. (SNNU); 1 male, Yunnan, Jinping, Mengla, Xinmeng, 450 m, May 3, 2013, Ma, Libin coll. (SNNU); 3 males, Yunnan, Mengla, Mengban, Hebianzhai, 855 m, May 23, 2013, Ma, Libin coll. (SNNU); 4 males, Yunnan, Mengla, Menglun, 690 m, May 28, 2013, Ma, Libin coll. (SNNU) ; 6 males, Yunnan, Cangyuan, Banlao, 1134 m, Jun. 5, 2013, Ma, Libin coll. (SNNU); 2 males, Yunnan, Hekou, 100 m, Jun. 7, 1982, Jin, Gentao coll. (SEM); 2 males and 1 female, Yunnan, Xishuangbanna, Menglun, 1000 m, Jun. 3, 2009, Liu, Xianwei coll. (SEM); 3 males and 2 females, Yunnan, Mengla, Yaoqu, Jun. 1, 2009, Ma, Libin coll. (NWAFU). **Vietnam**: 1 female, Tonkin, Jul. 1940, A. De Cooman coll. (SEM).

##### Distribution.

(Fig. 2). China (Yunnan, Guangdong), India, Vietnam.

##### Measurements.

BL 12.86–14.23; HW 3.54–3.75; PL 2.26–2.39; PW 4.12–4.56; FWL 9.13–9.68; HWL 7.45–8.23; MTL 3.24–3.46; CL 4.67–5.31; HTL 4.73–5.21; HLL 8.11–8.42; OL 7.28–7.64.

##### Diagnosis.

Body bicolored; head and legs often yellow or yellowish-brown, remainders always dark brown. Body size small for the genus. Both proximal and anterior notch of epiphallus arc-like and posterior notch almost right angular. Coloration of hind legs variable.

##### Description.

(Figs 1A–C, 8A–D, I). Body sized small for the genus, fusiform. Frons rounded. Median ocellus small, oval; lateral ocelli larger and rounded. Epistomal suture slightly upward convex and sometimes almost straight. Rostrum slightly widened. Scapus about half as wide as rostrum. Labrum rounded and slightly laterally widened, with apical margin arc-like, sometimes straight or with notch. Last segment of maxillary palpi rod-like, nearly as wide as third segment.

Disc of pronotum laterally widened and with hind margin slightly wider than fore margin; anterior margin broadly concave, posterior margin almost straight. Oblique veins three, the outmost one short and two internal of them longer and inclined; the top of them close each other. Diagonal vein straight. Chord veins three, the internal two veins extremely bent, connected at the bottom. Between the diagonal vein and the most internal chord vein armed with a transverse vein. The most internal chord vein linking with mirror by two transverse veins. Mirror small; the basal margin of mirror angle-like, dividing vein angular and the width of mirror nearly equal to the length. Field area short, close to the length of mirror, or slightly longer than mirror. Hind wings long and the uncovered portions longer than the half-length of forewings.

Fore tibiae with inner tympanum small and ovoid; the outer one large and oblong. Hind tibiae short, half the length of hind femur. Inner dorsal spurs of hind tibiae curved distally and longer than the outer ones. The length and number of dorsal spurs varied, while the basal spurs rather short, they numbered five or six of both the inner and outer; while spurs vary in length, the number of inner and outer spurs always 4:4. Inner apical spurs longer than outer ones. The median one of outer apical spurs longest and the remaining ones almost equal in length, and the bottom one of inner apical spurs shortest and the remaining ones in similar length. Inner dorsal spines of the first hind tarsus numbered 5–7 and outer ones numbered 7–9. Cercus thickness proximally and tapering. Subgenital plate simple and cucullate with acute apex.

***Genitalia*** (Fig. 8E–G). Lateral lobes of epiphallus large. In lateral view apex obtuse and slightly upward curved. Epiphallus without median lobe, hind margin broad arc-like in middle, proximal margin arcuate, similar to outer margin. In caudal view, ectoparamere with three ridges, two horizontal and one vertical; area around ridges dark, and central area whitish.

**Female** (Fig. 8B, D, J). Ovipositor short, arrow-like. Tegmina dark brown.

***Coloration*** (Fig. 8A–D, H). Head orange. Fore and median legs and hind tibiae yellowish-brown. Coloration of hind femur variable, either uniformly dark brown, or dark brown in middle and basal, and apical areas yellowish-brown or wholy yellowish-brown. Female’s forewings dark brown. Male’s forewings dark brown, but harp area and mirror light colored (these parts membranous and lucent). Cercus yellowish-brown with apical area dark brown.

##### Remarks.

The original description of *P.flavipes* does not mention genital characters. Xia et al. (1991) only pointed out that coloration of the hind femur and differences in the distance between the first and second oblique veins at the base could distinguish this species from *P.minor*. We showed that the distance at the base of oblique veins varies among individuals in *P.minor* (Figs 6, 8I). Moreover, the proportion of the black area on the hind femur also has a considerable amount of variation within *P.minor* (Fig. 7). The typical color scheme is 70% black (35% of the individuals), but more than 5% of the individuals have less than 10% or more than 95% of black area. The coloration of the hind femur is a continuous trait, and both extremes exist in *P.minor*. Hence, we consider that *P.flavipes* is a junior synonym of *P.minor*.

#### ﻿ Phonarellus ritsemae

Taxon classificationAnimaliaOrthopteraGryllidae

(Saussure, 1877)

2ED2606A-C085-5184-BF8A-C6907CB44447

**Figure 9.**
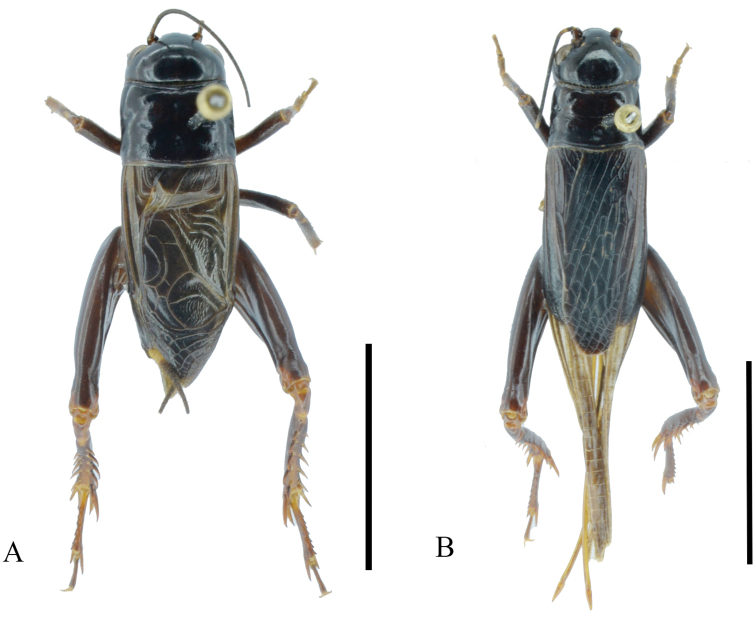
Body of *P.ritsemae***A** male **B** female. Scale bar: 10 mm.

**Figure 10.**
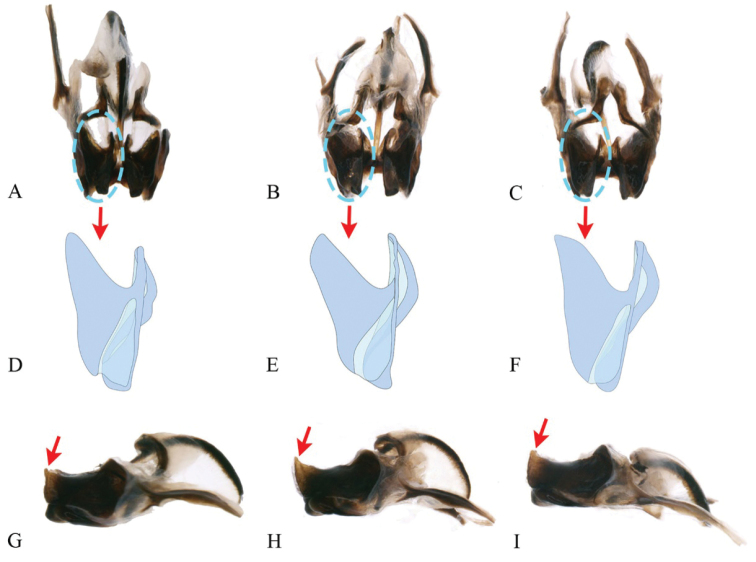
Intraspecific variation of genitalia of *P.ritsemae***A–C** genitalia of three individuals in ventral view **D–F** drawing of ectoparamere in ventral view **G–I** Genitalia of the same three individuals in lateral view (Note: The blue circles of the **A–C** graph point to the ectoparamere, highlighting variations among individuals; the red arrows in the **G–I** graph point to the teeth of epiphallic posterior).

**Figure 11.**
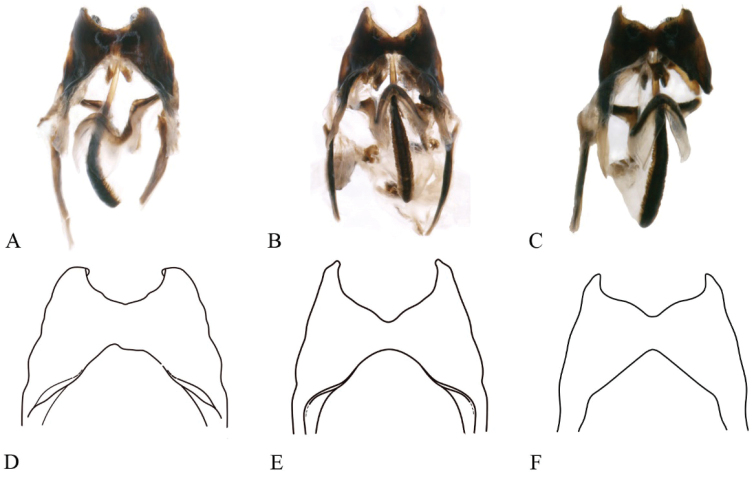
Intraspecific variation of genitalia in *P.ritsemae***A–C** photos of genitalia of three individuals in dorsal view **D–F** drawing of epiphallus of the same three individuals in dorsal view (Note: the drawing shows the notch variation)

Figs 1D , 9 , 10 , 11

Liogryllus ritsemae Saussure, 1877: 304; Chopard 1936: 4.
Acheta ritsemae : Shiraki 1930: 200.
Gryllus ritsemae : Hisumatsu 1952: 43.
Tartarogryllus ritsemae : Chopard 1961: 272; Randell 1964: 1582; Leroy 1966: 39; Chopard 1967: 73.
Phonarellus ritsemae : Yin and Liu, 1995: 138–139; Ichikawa et al. 2000: 260; Hollier et al. 2013: 515.

##### Holotype.

Type locality: Japan. Deposited at National Nature Historical Museum, Leiden, Netherlands (not examined).

##### Specimens examined.

**China**: 1 female, Yunnan, Mengla, Shangyong, Longmen, May 13, 2013, Ma, Libin (SNNU); 1 male, same location as before, May 14, 2013, Ma, Libin (SNNU); 1 male and 2 females, same location as before, May 18, 2013, Ma, Libin (SNNU); 1 male, Yunnan, Lvchun, Banpo, May 9, 2013, Ma, Libin. (SNNU); 1 female, Yunnan, Mengla, Yaoqu, May 25, 2013, Ma, Libin (SNNU); 1 female, Yunnan, Mengla, Menglun, May 28, 2013, Ma, Libin (SNNU); 9 males and 1 female, Guangdong, Shaoguan, Luoshanzhen, May 13, 2015, Zhang, Tao (SNNU); 8 males, Guangdong, Shenzhen, May 17, 2015, Zhang, Tao (SNNU); 1 male, Guangxi, Jingxi, Longbang, May 2, 2019, Ma, Libin and Zhang, Tao (SNNU); 1 male, Hong Kong, Damaoshan, May 9, 2018, Ma, Libin (SNNU); 1 male, Hong Kong, Fei’eshan, May 18, 2018, Ma, Libin and Peng, Zhong (SNNU).

##### Distribution.

China (Yunnan, Guangdong, Guangxi, Hong Kong), Japan..

##### Measurements (mm).

**Male (*N* = 22)**: BL 13.07–15.16; HW 3.76–4.15; PL 2.74–2.81; PW 4.05–4.25; FWL 7.88–10.05; HFL 8.97–9.62; HTL 4.74–5.75; **Female (*N* = 6)**: BL 15.65–16.48; HW 3.83–4.06; PL 2.51–2.75; PW 3.85–4.06; FWL 8.86–9.92; HFL 8.83–10.71; HTL 4.95–5.63.

##### Description.

***Male*** (Figs 1D, 9A). Body medium sized, fusiform. Head smooth, about as wide as pronotum. Occiput narrowed and convex. Vertex smooth and broad. Frontal rostrum rather wide, inverted trapezoid. Median ocellus small and shaped ovoid or semilunar. Lateral ocelli larger and located on both sides of the frontal rostrum. Eyes convex, about 1/4 length of head. Antennal socket triangular. Epistomal suture straight and twice as long as frontal rostrum. Postclypeus narrow. Labrum slightly convex and elliptical. The third of maxillary palpi longest, apical segments enlarge. Each section of labial palpi progressively longer. Disc of pronotum rectangular and middle groove unconspicuous. Forewings almost as long as abdomen. Hind femora about twice as long as tibiae; armed with five dorsal spurs on inner and outer dorsal margins and with three apical spurs on both sides (the ventral one shortest, and half-length of the others, the middle one with equal length of the dorsal), the outer apical spurs three (the middle one about twice longer than the others). Subgenital plate fusiform, at end narrowed and flattened.

***Genitalia*** (Figs 10, 11). Dark brown. Epiphallus with lateral lobes large with apex acute and slightly curved upward; without median lobe but in middle sinuate with angular notch. Epiphallic anterior margin angulates with straight lateral edges or with arc-like lateral edges (Fig. 11D–F). The bottom edge of the epiphallic lateral lobes extends horizontally at the posterior and downward at the anterior. Ectoparamere with three ridges (Fig. 10D–F), a pair of horizontal ones and a vertical one; area around ridges dark, and central area whitish lucent.

**Female** (Fig. 9B). Resembles male but larger. Ovipositor as long as forewings. Hindwings white and of equal length as forewings.

***Coloration*** (Figs 1D, 9). Body dark brown. Head, pronotum black-brown. Femora reddish brown.

##### Remarks.

Yin and Liu (1995) recorded this species from Shanghai, China, but there was no detailed description of this species. We collected a large number of specimens from Yunnan and Guangdong, China and identified them as *P.ritsemae*. We observed a few traits with intraspecies variation: (1) the posterior teeth of the epiphallus are variable among individuals in lateral view (as shown in Fig. 10G–I); (2) the ectoparamere can have different shapes in ventral view (as shown in Fig. 10D–F). (3) the notch at hind margin of the epiphallus can be rather wide or slightly contracted, and the ventral margin can be broad or angular in dorsal view (as shown in Fig. 11D–F); and (4) the epiphallic anterior margin can be angle-like with straight lateral edges or broad and arc-like in dorsal view (as shown in Fig. 11D–F).

## ﻿Discussion

Species delimitation is the foundation for biodiversity research, and finding robust and reliable characters for species identification is crucial for taxonomy. However, one difficulty faced by morphology-based species delimitation is distinguishing intraspecific variation from interspecific difference. When only a few specimens are accessible, we might mistake variation within species as species difference. Then, species identification might be unstable, leading to confusion in future taxonomic work. Here, we examined a large number of specimens in two cricket genera *Gymnogryllus* and *Phonarellus*. With multiple specimens, we revealed a considerable amount of morphological variation within species.

Notably, both genera possess intraspecific variation in male genitalia, features which are primary characters for species identification in insects (Song 2009) and crucial for cricket taxonomy (Alexander and Otte 1967). The most famous hypothesis to explain the diversity in male genitalia between species is the lock-and-key hypothesis: male genitalia need to structurally match female genitalia of the same species (Dufour 1844). However, in this case, there should be minimal variation in male genitalia within species (Andrade et al. 2009). Comparing specimens collected at the same time from the same site, we found that the end of the epiphallus of *G.odonopetalus* is flattened or grooved to varying degrees among individuals. The apical epiphallus notch of *P.ritsemae* could expand outward or contract, and the depth of the notch varyable among individuals. These genitalic features are probably involved in holding and fixing the female during mating (Edvardsson and Canal 2006). For example, the ectoparamere is movable, and epiphallic teeth can provide a good grasping function and ensure the stability of the mating process. However, in *G.odonopetalus*, epiphallic teeth could be stubby or slender in shape, and the size of the space between the teeth varies in different individuals. These intraspecific variations we observed contradict the lock-and-key hypothesis. Instead, it is concordant with the hypothesis of sexual selection, which predicts a larger variation within species. More quantitative morphometric analyses are needed to test alternative hypotheses further.

We also observed intraspecific polymorphism with regard to body color in *P.minor*. Body color plays an essential role in adapting to environmental changes, resisting diseases, and avoiding predators (Ahnesjo and Forsman 2006). Varying the proportion of black color on body surface might be related to temperature regulation (Clusella-Trullas et al. 2007) and minimizing the likelihood of being founded by natural enemies (Forsman and Appelquist 1998). In this species, the distribution of the black color proportion on the hind femora is a continuous, bell-shaped distribution. It probably is a quantitative trait under stabilizing selection.

Based on our discovery of intraspecific variation, we considered some diagnostic features previously used as characters for separating species in these two genera invalid. The new species checklist showed five synonymus (*G.yunnanensis*, *G.striatus*, *G.longus*, *G.tumidulus* and *P.minor*), which reduces the number of Chinese species of the genera *Gymnogryllus* and *Phonarellus* to four and three, respectively. Our work highlights the importance of extensive specimen collection and considering intraspecific variation in species identification.

## Supplementary Material

XML Treatment for Gymnogryllus

XML Treatment for Gymnogryllus odonopetalus

XML Treatment for Phonarellus

XML Treatment for Phonarellus minor

XML Treatment for Phonarellus ritsemae

## Supplementary materials

Supplementary material 1 The distance between the first and second oblique veins at the base varies of 42 specimens of the *P.minor* Data type statistical data https://binary.pensoft.net/file/770027This dataset is made available under the Open Database License (http://opendatacommons.org/licenses/odbl/1.0/). The Open Database License (ODbL) is a license agreement intended to allow users to freely share, modify, and use this Dataset while maintaining this same freedom for others, provided that the original source and author(s) are credited.Ning Wang, Huateng Huang, Li-Bin Ma

Supplementary material 2 The area to total area ratio of the black area of the posterior femora of 42 specimens of the *P.minor* Data type statistical data https://binary.pensoft.net/file/770028This dataset is made available under the Open Database License (http://opendatacommons.org/licenses/odbl/1.0/). The Open Database License (ODbL) is a license agreement intended to allow users to freely share, modify, and use this Dataset while maintaining this same freedom for others, provided that the original source and author(s) are credited.Ning Wang, Huateng Huang, Li-Bin Ma
